# Editorial: chemicals and bioproducts from biomass

**DOI:** 10.1186/s13068-016-0637-4

**Published:** 2016-10-31

**Authors:** James C. du Preez

**Affiliations:** Department of Microbial, Biochemical and Food Biotechnology, University of the Free State, P.O. Box 339, Bloemfontein, 9300 South Africa

The current driving force for the development of “clean” technologies for the production of fuels and chemicals from renewable resources stems mainly from environmental concerns, which have overshadowed concerns regarding the depletion of fossil fuel reserves and the price instability of crude oil. To accommodate the increasing interest in biotechnology for the production of chemicals, *Biotechnology for Biofuels* (*BfB*) took the decision to widen its scope to also include articles on the biological production of chemicals and bioproducts derived from biomass and CO_2_. Whereas a large proportion of the articles in *BfB* still deal with the pretreatment, hydrolysis and bioconversion of lignocellulosic biomass to ethanol, the journal also has published a number of interesting articles related to bio-based chemicals and other bioproducts. This retrospective thematic issue assembles recent articles since 2015 to highlight these areas of research. An interesting observation when compiling this thematic issue was that of these 32 articles, 11 originated from China—by far the most from any one country.

The topics covered by these selected articles are rather diverse, with feedstocks ranging from sugars to syngas (a mixture of CO, H_2_ and CO_2_), while the products include various chemicals and potential alternative biofuels such as acetate, *n*-butanol, 1-propanol, 1,2-propanediol, lipids, short- and medium-chain monocarboxylic acids and their corresponding alcohols, α-linolenic acid, succinic acid, glycolic acid, 3-hydroxypropionic acid, geraniol, chitin, isoprene, alkanes, 1-alkenes and biohythane (a mixture of biomethane and biohydrogen) produced from waste sludge using microbial electrolysis cells. The increasing number of articles dealing with the use of glycerol as feedstock for the production of bioproducts such as malic acid, 1,3-propanediol, 2,3-butanediol, triacylglycerides, erythritol and citric acid is encouraging, since valorisation of the huge amounts of crude glycerol generated as the main by-product of biodiesel production by transesterification of animal fats and plant oils has become imperative. Also included in this collection of articles is a review by Luo et al. [1] on the microbial synthesis of poly-γ-glutamic acid, a bio-based chemical that is already widely used in several industries and the production of which by microbial fermentation is deemed cost-effective.

Apart from conventional bioprocesses such as anaerobic digestion and fermentation, a significant increase was noted in the number of articles involving metabolic engineering and also protein engineering aimed at the production of chemicals and alternative biofuels. The genetically tractable *Escherichia coli* still featured as the favoured host microorganism for metabolic engineering. Yao et al. [2] investigated the co-metabolism of glycerol and glucose to determine the metabolic potential of a genetically engineered *E. coli* strain as a platform for the production of biofuels and chemicals. For the production of geraniol, an acyclic monoterpene alcohol that finds application in the perfume and other industries, Liu et al. [3] overexpressed several key pathway genes in *E. coli*. The production of *n*-butanol, which has excellent qualities for an alternative fuel, was investigated by Saini et al. [4] through engineering of the central metabolism of *E. coli*. A novel approach to the biosynthesis of long-chain 1-alkenes from low-cost triacylglycerols as a “drop-in” biofuel is described in the article by Yan et al. [5] on the development of a tandem biotransformation process incorporating cell-free systems (purified enzymes or cell-free extracts) and engineered *E. coli* whole cells. Protein engineering of key enzymes offers the prospect of tailoring biofuel formulations to desired specifications [6].

This selection of articles includes reports on metabolic engineering also of other microorganisms aimed at a variety of bioproducts. Extensive engineering of the workhorse of industrial biotechnology, *Saccharomyces cerevisiae*, enabled this yeast to produce fatty acid short- and branched-chain alkyl esters, including ethyl, isobutyl, isoamyl and amyl esters from endogenously synthesised fatty acids and alcohols at a high titre [7]. For the efficient biological production of chemicals and biofuels, the toxicity of the product towards the host cells can hamper efficient production. This aspect was addressed by Ling et al. [8], who improved the tolerance of *S. cerevisiae* to alkanes by engineering transcription factors. Also featured in this thematic issue is an article on the metabolic engineering of a non-conventional yeast, *Yarrowia lipolytica*, for enhanced glycerol assimilation and the production of erythritol and citric acid [9]. By targeting endogenous enzymes in *Corynebacterium glutamicum*, Siebert and Wendisch [10] improved the production of 1,2-propanediol, a versatile bulk chemical. They also succeeded in engineering this bacterium to produce 1-propanol, albeit at a low yield. Other examples of metabolic engineering for the bioproduction of chemicals are mentioned below.

The production of certain chemicals via a biotechnological route has considerable potential. A case in point is 2,3-butanediol, which as a commodity chemical has numerous industrial applications. Cho et al. [11] used glycerol as sole carbon source for 2,3-butanediol production by an engineered strain of *Klebsiella oxytoca*, reaching a high concentration of 131.5 g L^−1^ with little by-product formation, while Yang et al. [12] engineered *Zymomonas mobilis* for the efficient utilisation of biomass-derived mixed C5/C6 sugar streams. Optically pure 2,3-butanediol, on the other hand, is an excellent precursor for the asymmetric synthesis of high-value chiral chemicals, but the chemical processes for its production are expensive and yield a product of a low optical purity. However, the biotechnological route now seems an attractive alternative for obtaining pure isomers. Chu et al. [13] designed a metabolic pathway in *E. coli* for the direct production of (2*S*,3*S*)-2,3-butanediol from glucose, and Qiu et al. [14] described the production of the *meso*-2,3-butanediol isomer at a high titre with a purity greater than 99 % using a metabolically engineered strain of *Bacillus licheniformis*.

The economic viability of bioprocesses using renewable feedstocks, especially lignocellulosic biomass, often is a challenge. Recognising that an integrated biorefinery approach, where value-added chemicals are produced in conjunction with biofuels, offers an economic advantage, Bradfield et al. [15] investigated the continuous production of succinic acid from the xylose-enriched fraction of a corn stover hydrolysate stream using immobilised *Actinobacillus succinogenes* cells. Also using xylose-rich hemicellulosic hydrolysate as feedstock, Alkim et al. [16] achieved a high yield of glycolic acid through pathway engineering of *E. coli*. Both succinic and glycolic acids have numerous industrial applications. Gao et al. [17] proposed a strategy for the integration of biodiesel and succinic acid production using an engineered strain of *Y. lipolytica*. The biorefinery approach has also been applied to fully utilise animal wastes for the production of methane with chitin as by-product [18].

Microalgae, which by definition include small eukaryotic algae and prokaryotic cyanobacteria, have the advantage of being able to use solar energy and CO_2_ as carbon source. Although their use for the production of fuels and high-value chemicals has potential, commercial implementation suffers from the constraints of yield, productivity and cost. Flassig et al. [19] used modelling based on dynamic flux balance analysis to significantly improve β-carotene accumulation in *Dunaliella salina*. In a novel approach to enhance lipid production, Miranda et al. [20] used strains of a genetically modified cyanobacterium, *Synechocystis*, in a synergistic co-culture with a filamentous fungus. Following the integrated biorefinery concept to improve the economics of microalgal biodiesel production, Parsaeimehr et al. [21] investigated the co-accumulation of lipids and α-linolenic acid by *Chlorella sorokiniana*.

This compilation of articles is a mere sample of the dramatic developments over the past few years in the production of bio-based chemicals. The recent and anticipated future advances in genomics technologies, bioinformatics, systems biology and synthetic biology promise to revolutionise industrial biotechnology, leading to economically efficient carbon-neutral bio-industries producing a wide range of bulk and high-value chemicals.
